# Short-Chain Fatty Acids and the Gut–Retina Connection: A Systematic Review

**DOI:** 10.3390/ijms26062470

**Published:** 2025-03-10

**Authors:** Elena Ciurariu, Andreea-Talida Tirziu, Norberth-Istvan Varga, Bogdan Hirtie, Alexandru Alexandru, Cristiana-Smaranda Ivan, Laura Nicolescu

**Affiliations:** 1 Department of Functional Sciences, Physiology, Centre of Immuno-Physiology and Biotechnologies (CIFBIOTEH), “Victor Babeş” University of Medicine and Pharmacy, Eftimie Murgu Square, No. 2, 300041 Timisoara, Romania; ciurariu.elena@umft.ro; 2 Doctoral School, Department of General Medicine, “Victor Babeş” University of Medicine and Pharmacy, Eftimie Murgu Square, No. 2, 300041 Timisoara, Romania; norberth.varga@umft.ro (N.-I.V.); bogdan.hirtie@umft.ro (B.H.); 3 Department of General Medicine, “Victor Babeş” University of Medicine and Pharmacy, Eftimie Murgu Square, No. 2, 300041 Timisoara, Romania; alexandru.alexandru@student.umft.ro (A.A.); smaranda.ivan@student.umft.ro (C.-S.I.); 4 Doctoral School, Faculty of Medicine, “Vasile Goldis” Western University, Bulevardul Revolutiei 94, 310025 Arad, Romania; laura_dsp@yahoo.com

**Keywords:** gut–retina axis, short-chain fatty acids, SCFA, gut microbiota, ocular health, diabetic retinopathy, age-related macular degeneration, glaucoma

## Abstract

The interplay between gut microbiota and retinal health, known as the gut-–retina axis, has gained increasing attention in recent years. Short-chain fatty acids (SCFAs), metabolites produced by gut microbiota, have been identified as key mediators of gut–retina communication. This systematic review explores the role of SCFAs in retinal health and their potential impact on the development and progression of retinal diseases, such as diabetic retinopathy (DR), age-related macular degeneration (AMD), and glaucoma. A literature search was conducted across multiple databases, including PubMed, Google Scholar, and Science Direct, to identify studies published between 2014 and December 2024. Studies were included if they investigated the effects of SCFAs on retinal structure, function, or disease pathogenesis in animal models or human subjects. The review included 10 original articles spanning both preclinical and clinical studies. Evidence suggests that SCFAs play a crucial role in maintaining retinal homeostasis through anti-inflammatory and neuroprotective mechanisms. Dysbiosis of the gut microbiota, leading to altered SCFA production, was associated with increased retinal inflammation, oxidative stress, and vascular dysfunction. Furthermore, reduced SCFA levels were linked to the progression of retinal diseases, such as diabetic retinopathy and age-related macular degeneration. Modulation of gut microbiota and SCFA levels through dietary interventions or probiotics may represent a novel therapeutic strategy for preventing or managing retinal diseases. Further research is needed to elucidate the precise molecular mechanisms underlying SCFA-mediated retinal protection and to evaluate the efficacy of targeted therapies in clinical settings.

## 1. Introduction

Retinal diseases, including age-related macular degeneration (AMD), diabetic retinopathy (DR), and glaucoma, represent a significant global health burden, collectively standing as leading causes of irreversible vision loss and blindness worldwide [1,2,3]. AMD alone affects millions of individuals, predominantly those over 60 years old, with projections indicating a substantial increase in prevalence in the coming decades [3,4]. Similarly, DR constitutes a major complication of diabetes, impacting a considerable portion of the diabetic population and contributing significantly to vision impairment [5]. Glaucoma, often referred to as the “silent thief of sight”, adds to this burden, causing progressive and irreversible damage to the optic nerve [6,7]. Current therapeutic approaches for these diseases, such as anti-VEGF (vascular endothelial growth factor) therapy for neovascular AMD, complement inhibitors like pegcetacoplan for dry AMD, laser photocoagulation for DR, and intraocular pressure-lowering medications for glaucoma, present limitations [8,9,10]. While these treatments can slow disease progression and, in some cases, improve vision, they often fail to address the underlying causes of retinal diseases. This highlights the pressing need for a deeper understanding of the pathophysiological mechanisms involved and for novel therapeutic strategies that target the root causes of these debilitating conditions. In recent years, the gut microbiota, a complex community of microorganisms residing in the human gastrointestinal tract, has emerged as a key player in human health and disease [11,12,13,14]. This intricate ecosystem, once considered merely a bystander in human physiology, is now recognized as a crucial modulator of various host processes, including metabolism, immunity, and even neurological function.

The recognition of the gut microbiota’s profound influence on systemic health has spurred research into its potential role in extra-intestinal organs, including the eye. This has led to the conceptualization of the “gut–retina axis”, a bidirectional communication pathway linking the gut microbiome and the retina [12,13,14,15,16,17]. New studies suggest that imbalances in the gut microbiota, termed dysbiosis, can contribute to the pathogenesis of retinal diseases through several mechanisms. These include the disruption of intestinal barrier integrity, leading to increased translocation of bacterial products like lipopolysaccharide (LPS) into the circulation, thereby triggering systemic inflammation, which is known to impact retinal health. An emerging body of literature focuses on the role that short-chain fatty acids (SCFAs), the main metabolites produced by the gut microbiota through the fermentation of dietary fibers, play in human health [18,19,20,21,22]. These molecules, primarily acetate, propionate, and butyrate, produced in varying ratios by microbial fermentation, are not only a crucial energy source for colonocytes but also exert potent anti-inflammatory, immunomodulatory, and neuroprotective effects. SCFAs can interact with specific G-protein coupled receptors (GPCRs), such as GPR41, GPR43, and GPR109A, and inhibit histone deacetylases (HDACs), thereby influencing gene expression and cellular function in various tissues, including the retina [23,24,25]. Notably, GPR43 and GPR109A are expressed in retinal pigment epithelium and microglia, respectively, while immune cells like macrophages bear these receptors, hinting at direct SCFA–retina interactions. Given the high metabolic demands and unique immune environment of the retina, it is plausible that alterations in SCFA levels and signaling could significantly impact retinal homeostasis and contribute to disease pathogenesis. Aging further complicates this ecosystem, often reducing microbial diversity and SCFA production—shifts that may exacerbate retinal diseases tied to older age. An increasing number of studies have reported alterations in gut microbiota composition and SCFA levels in patients with retinal diseases, further supporting the existence of a gut–retina axis [26,27]. Moreover, experimental evidence from animal models has demonstrated that manipulating the gut microbiota or administering SCFAs can influence retinal inflammation, neovascularization, and neuronal survival.

Despite the growing body of evidence supporting the involvement of the gut microbiota and SCFAs in retinal health, many questions remain unanswered. Therefore, this systematic review aims to synthesize the current evidence linking the role that SCFAs play on retinal diseases. This review focuses on studies investigating alterations in gut microbiota composition and SCFA levels in these conditions, as well as the effects of interventions targeting the gut–retina axis, such as SCFA supplementation, dietary modifications, and microbiota modulation. Furthermore, it aims to explore the implicated molecular mechanisms, including the roles of SCFA receptors, HDAC inhibition, and inflammatory pathways. By comprehensively analyzing the available data, this review seeks to provide a deeper understanding of the gut–retina axis, highlight the potential of SCFAs as therapeutic agents, and identify key knowledge gaps and future research directions in this rapidly evolving field.

## 2. Materials and Methods

### 2.1. Search Strategy

**Figure 1:**
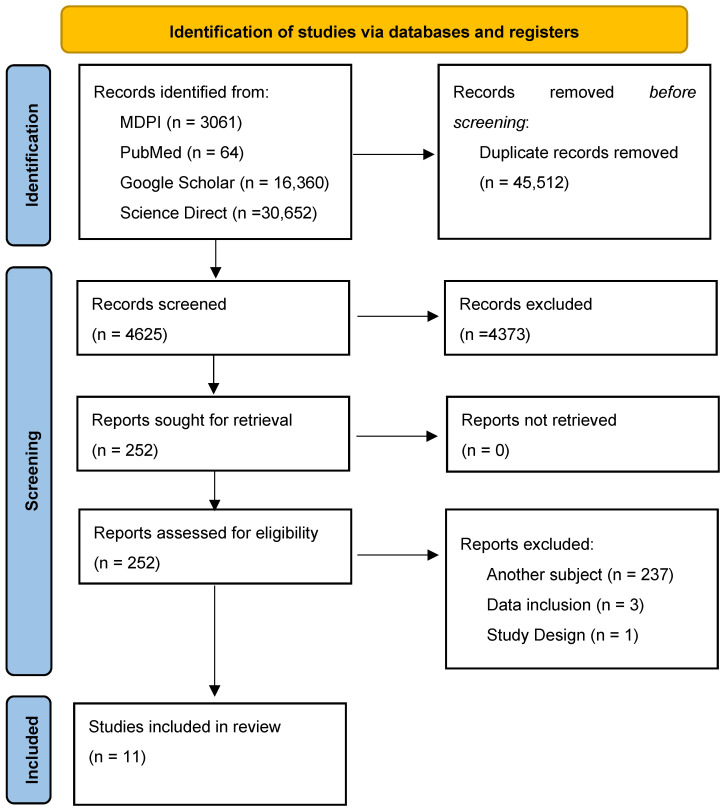
PRISMA flowchart of the study selection process.

For the purpose of this systematic review, we utilized standard operating principles as outlined in the Preferred Reporting Items for Systematic reviews and Meta-Analyses (PRISMA) reporting guidelines [28] (Figure 1).

The databases utilized were MDPI, PubMed, Google Scholar, and Science Direct.

The following search strategy was applied:

- Keyword Search: We used specific keywords and phrases, utilizing the MeSH feature of PubMed, such as “short chain fatty acids”, “butyrate”, “nervous system cells”, “neurons”, “ganglion cells”, “amacrine cells”, “bipolar cells”, “horizontal cells”, “photoreceptors”, “astrocytes”, “microglia”, “Müller cells”, “retina”, “macula lutea”, “retinal diseases”, “molecular mechanisms”, “epigenetic regulation”, “histone acetylation”, “histone deacetylase inhibitors”, “gene expression”, “signal transduction”, “immune modulation”, “oxidative stress”, “anti-inflammatory”, “neuroprotection”, “cell survival”, “photoreceptor survival”, “gut-brain axis”, “epigenetic mechanisms”, “inflammation”, “histone deacetylase inhibition”, “acetyl-CoA metabolism”, “retinal ganglion cells”, “retinal degeneration”, “macular degeneration”, “dysbiosis”, “intestinal microbiology”, “gut microbiota”, “microbiome”.
- Boolean Operators: Operators like AND, OR, and NOT were used to refine search results (e.g., (“short chain fatty acids” OR “butyrate”) AND (“retina” OR “macula lutea”)).
- Filters Used: Articles in the English language were used, and the rest were discarded. Abstracts were reviewed to select studies that met the inclusion criteria. Only articles that had the full text available were utilized for review and citation. Any duplicates were then removed.
- Snowballing Approach: To ensure a comprehensive review of the literature, we employed the snowballing technique. This involved systematically examining the reference lists of key articles identified during the initial search to uncover additional studies pertinent to our research topic. By tracing these references, we aimed to identify relevant publications that may not have been captured in our primary search strategy, thereby enhancing the breadth and depth of our review.

Our review included publications from the last 10 years to ensure that the most current and relevant research was considered. Utilizing the snowballing method, highly cited papers were sought after reviewing the bibliography of the relevant published literature in the hope of citing the source papers in most instances. This review was not prospectively registered in any database.

### 2.2. Study Selection and Data Analysis

In order to ensure the quality and reliability of the included sources, two authors independently assessed the publications. Any disagreements were resolved through discussion or, when necessary, by consulting a third author. For the screening process, two independent reviewers (A.A. and I.C.-S.) evaluated all records for eligibility. The inter-rater reliability, measured by Cohen’s Kappa, was 0.83, indicating a very high level of agreement. Discrepancies were addressed through consensus or, if unresolved, by involving a third reviewer (N.-I.V.).

The inclusion criteria were as follows: (1) studies with abstracts relevant to the effects of SCFA on retina; (2) full-text articles published in English from 2014 to 2024; and (3) original research articles with the following study designs: animal experimental studies and human studies (observational cohort studies and controlled trials).

The exclusion criteria were as follows: (1) studies focusing on topics irrelevant to our research objective; (2) articles published in a language other than English, with no translation available (e.g., French, Spanish, Chinese); (3) studies not published in a peer-reviewed journal; (4) research resources without available full-text (abstract only); and (5) inappropriate study designs (e.g., reviews, case reports, etc.).

After the final list of selected studies was agreed upon, two separate reviewers independently extracted the relevant data from the included studies and systematically organized it into a table, ensuring a methodological approach for our analysis. The following data were extracted: study ID (first author and year of publication), study design, investigated species, sample size, sample type, analysis methodology, dominant bacterial genera, SCFAs in analysis, and the statistical significance.

## 3. Results

### 3.1. Overview of Included Studies

An initial search was conducted across multiple databases, including MDPI, PubMed, Google Scholar, and ScienceDirect, using the specified keywords in an advanced search with a 10-year timeframe restriction in order to present the most actual and relevant findings, yielding a total of 50,137 studies. After removing 45,512 duplicates, 4625 articles remained and were screened by two independent reviewers to ensure eligibility. Of these, 4374 articles were excluded for reasons including (1) lack of relevance to SCFA and the retina–gut axis, (2) absence of clinical outcome data related to the retina–gut connection, (3) publication in a non-peer-reviewed journal, (4) unavailability of an English translation, or (5) use of a systematic review, meta-analysis, scientific letter or abstract for scientific presentation, instead of original research. Following this initial screening, 251 studies were considered eligible and proceeded to a full-text assessment. During this second round, previous and additional exclusion criteria, such as irrelevant subject focus, incorrect data inclusion, or inappropriate study design, were applied. The study selection process is summarized in Figure 1.

This study selection process resulted in the inclusion of 11 studies investigating the interplay between short-chain fatty acids, the gut microbiota, and retinal diseases, with a particular focus on the gut–retina axis [29,30,31,32,33,34,35,36,37,38,39]. The majority of the experimental studies utilized rodent models (mice and rats), including C57BL/6J mice, Sprague Dawley rats, and Wistar rats, to investigate the effects of SCFAs on retinal health [29,30,31,32,33,34,35,36]. These studies incorporated both in vivo and in vitro approaches. In vitro experiments often used human cell lines, such as human umbilical vein endothelial cells (HUVECs) and the ARPE-19 retinal pigment epithelial cell line, to explore the cellular and molecular mechanisms underlying SCFA action. The human studies included cross-sectional analyses [38], a longitudinal cohort [39], and a randomized clinical trial [37], involving participants with glaucoma, diabetic retinopathy (DR), or neovascular age-related macular degeneration (nAMD), as well as healthy controls. Tabel 1 offers a summary of the included studies.

A wide range of methodologies was employed across the included studies. Gut microbiota composition was primarily assessed using 16S rRNA gene sequencing, with some studies also incorporating metagenomic sequencing [32,35,39]. SCFA levels were quantified using Gas Chromatography–Mass Spectrometry (GC-MS) or Liquid Chromatography–Mass Spectrometry/Mass Spectrometry (LC-MS/MS). Gut microbiota profiling leaned on 16S rRNA sequencing for its cost-effective taxonomic snapshot, though it skips functional insights, while metagenomic sequencing, used in some studies, delivers richer gene-level data with a higher cost and complexity. Other techniques included Western blotting, flow cytometry, enzyme-linked immunosorbent assay (ELISA), quantitative real-time PCR (qPCR), RNA sequencing, histology, immunohistochemistry, Optical Coherence Tomography (OCT), electroretinography (ERG), and specialized assays like the chorioallantoic membrane (CAM) assay (see Table 1).

As for interventions and exposures, some studies investigated the effects of direct SCFA administration, primarily focusing on sodium butyrate, either through oral gavage [35,36] or in drinking water [31]. Other studies used dietary interventions like high-fiber diets [34] or prebiotic supplementation, known to indirectly influence SCFA production by the gut microbiota. One study employed a unique approach using micronutrient supplementation containing lutein, zeaxanthin, and saffron [37]. Additionally, one study investigated the impact of oral metformin administration, a drug known to alter gut microbiota composition, on choroidal neovascularization (CNV) in mice [33].

The included studies employed a diverse range of outcome measures to comprehensively assess the interplay between the gut microbiota, SCFAs, and retinal health. Retinal outcomes encompassed structural evaluations, such as neovascularization (quantified by CNV lesion size), retinal thickness, and retinal cell loss, as well as functional assessments, including visual acuity and electroretinography (ERG) parameters. Inflammatory responses in the retina were evaluated through microglial activation and molecular analyses, including retinal gene expression. Microbiota outcomes focused on characterizing the composition and diversity of the gut microbiome, quantifying specific bacterial taxa, and predicting functional pathways. Metabolite outcomes primarily involved quantifying fecal and/or plasma levels of short-chain fatty acids. No uniform SCFA panel emerged—most studies targeted acetate, propionate, and butyrate, though some branched into valerate or caproate, reflecting methodological diversity. Systemic outcomes included measurements of inflammatory cytokine levels (e.g., IL-6, TNF-alpha), blood glucose, lipid profiles, and body weight, reflecting the broader physiological impact of the gut–retina axis.

### 3.2. Alterations in SCFA Levels in Retinal Diseases

The studies included in this review investigated alterations in SCFA levels in the context of retinal diseases, providing evidence for the involvement of these gut-derived metabolites in the gut–retina axis. Notably, studies focusing on diabetic retinopathy (DR) consistently reported reduced systemic levels of certain SCFAs. Specifically, Qin et al. (2024) [39] observed lower plasma acetate and butyrate in DR patients compared to diabetic controls without retinopathy. Similarly, Huang et al. (2023) [36] found that STZ-induced diabetic mice exhibited significantly decreased plasma levels of butyric acid, 4-methylvaleric acid, and caproic acid compared with the control group, indicating that diabetes itself may contribute to reduced systemic SCFA levels, being in line with the findings of Shen et al. (2022) [34], who also found decreased levels of acetate, propionate, and butyrate in STZ-treated animals. Furthermore, Chen et al. (2022) [32] found increased levels of acetate, propionate, butyrate, isobutyrate, valerate, isovalerate, and caproate in the fecal and blood samples of patients with glaucoma. In contrast, Baldi et al. (2024) [37] reported a significant reduction in total fecal SCFA levels in patients with neovascular age-related macular degeneration (nAMD) compared to healthy controls. The reduction in systemic SCFA levels observed in DR and nAMD suggests a potential link between diminished SCFA availability and the pathogenesis of these retinal diseases.

In a similar vein, Rowan et al. (2017) [29] found that mice on a high-glycemic diet showed reduced fecal butyrate and acetate levels alongside worsened retinal damage in an AMD model, while a low-glycemic diet restored these SCFAs and eased photoreceptor loss. This aligns with Qin et al.’s (2024) [39] findings in DR patients, where diminished acetate and butyrate tracked with disease progression, suggesting a broader pattern of SCFA depletion in retinal pathology. Together, these studies hint that diet-driven dysbiosis could amplify retinal stress across conditions like AMD and DR.

**Table 1:** Summary of Included Studies; POAG = Primary Open Angle Glaucoma; SEM = Scanning Electron Microscopy; OCT = Optical Coherence Tomography; HPLC = High-Performance Liquid Chromatographic method; STZ = Streptozotocin; GC-MS = Gas Chromatography–Mass Spectrometry; ERG = electroretinography; NaBu = sodium butyrate; LA = linoleic acid; ALA = alpha-linolenic acid; LC-MS = Liquid Chromatography–Mass Spectrometry; nAMD = neovascular age-related macular degeneration; HG/LG diet-AMD = high-/low-glycemic diet-induced age-related macular degeneration; Laser–CNV = laser-induced choroidal neovascularization; LPS-uveitis = lipopolysaccharide-induced uveitis; POAG = Primary Open-Angle Glaucoma (human patients, mouse model unspecified); STZ-T1DM = Streptozotocin-induced Type 1 Diabetes Mellitus.

Study	Study Design	Species Investigated	Sample Size (n)	Model	Sample Type	Analysis Methodology	Dominant Bacterial Genera	SCFA	Statistical Significance (*p*-Value)
Rowan et al., 2017 [29]	Experimental (in vivo)	C57BL/6J mice	60	HG/LG diet-AMD	Fecal, retinal tissue	16S rRNA sequencing, metabolomics, histology	*Bacteroides*, *Prevotella*	Butyrate and acetate increased with a high-fiber diet	<0.05
Xiao et al., 2020 [30]	Experimental (in vitro and in vivo)	C57BL/6J mice, HUVECs	100 mice (in vivo), several replicates (in vitro)	Laser–CNV	Choroid tissue,	Western blot, Choroid Sprouting Assay, RT-PCR, Immunofluorescence Histochemistry	NA	Dose-dependent reduction in neovascularization and SCFA-modulated pathways	<0.05
Chen et al., 2021 [31]	Experimental (in vitro and in vivo)	C57BL/6J mice	~50 mice (in vivo), multiple replicates (in vitro)	LPS-uveitis	Retinal tissues, RAC culture	GC-MS, ELISA, Western blot, flow cytometry	NA	SCFA (butyrate, propionate) inhibited inflammatory cytokine production	<0.05
Chen et al., 2022 [32]	Experimental (human and mouse models)	C57BL/6J mice, human POAG patients	~50 mice, 22 patients	POAG	Fecal, serum, retinal tissues	Metagenomics, GC-MS, 16S rRNA sequencing	*Dysgonamonadaceae* (enriched in POAG), *Barnesiellaceae* (enriched in controls)	SCFA increase observed in POAG; reduction post-antibiotic treatment	<0.05
Dos Reis et al., 2022 [33]	Experimental (in vitro and in vivo)	Wistar rats, ARPE-19 cells, CAM	18 rats, multiple replicates	Laser–CNV	Retinal tissue, CAM, cultured cells	SEM, OCT, CAM assay, HPLC, histopathology	NA	Controlled release of NaBu for 35 days with antiangiogenic effect	<0.05
Shen et al., 2022 [34]	Experimental (in vivo, human data included)	Sprague Dawley rats, human subjects	20 rats; 20 human subjects	STZ-T1DM	Retinal, plasma, vitreous fluid	GC-MS, ELISA, 16S rDNA sequencing, histopathology	*Ruminococcaceae*, *Prevotellaceae*, *Alloprevotella*, *Bifidobacterium pseudolongum*	Yes (STZ-induced T1DM reduced SCFAs; restored with LA and ALA)	<0.05
Zhang et al., 2023 [35]	Experimental (in vivo)	C57BL/6J mice	47 mice	Laser–CNV	Retinal, fecal samples	RNA sequencing, 16S rRNA sequencing, LC-MS	*Akkermansia*, *Bifidobacterium*	Increased SCFA levels (butyrate, propionate) in metformin group	<0.05
Huang et al., 2023 [36]	Experimental (in vivo)	C57BL/6J mice	~20 mice	STZ-T1DM	Plasma, fecal, retinal tissue	OCT, HE staining, electroretinography, LC-MS/MS, 16S rRNA sequencing	*Dubosiella*, *Ileibacterium*, *Lachnospiraceae*	Butyric acid, caproic acid, and 4-methylvaleric acid increased with NaB	<0.05
Baldi et al., 2024 [37]	Randomized clinical trial	Human	45	N/A	Stool, plasma	16S rRNA sequencing, GC-MS, OCT	*Faecalibacterium*, *Lachnospiraceae*	Total SCFA levels reduced in nAMD group compared to HC; partially restored with intervention	<0.05
Vergroesen et al., 2024 [38]	Observational	Human	1472	N/A	Stool samples	16S rRNA sequencing, meta-analysis	*Butyrivibri*, *Caproiciproducens*, *Clostridium* *sensu stricto 1*	Decreased butyrate-producing taxa in glaucoma group	<0.05
Qin et al., 2024 [39]	Cross-sectional and longitudinal cohort	Human	161	N/A	Stool, plasma, PBMCs	16S rRNA sequencing, GC-MS, transcriptomics	*Butyricicoccus*, *Ruminococcus torques*	Acetate and butyrate reduced in DR patients	<0.05

While Chen et al. (2022) [32] found elevated SCFAs in blood and feces of glaucoma patients, suggesting systemic reach, direct evidence of SCFAs in the ocular environment remains limited. In addition to this, other studies investigated the levels of SCFAs in the context of glaucoma, further supporting the importance of these metabolites in this disease. Vergroesen et al. (2024) [38] identified a lower abundance of butyrate-producing taxa in the gut of glaucoma patients, suggesting a potential link between gut dysbiosis and reduced butyrate availability in glaucoma. While the precise mechanisms by which SCFAs influence retinal health remain to be fully elucidated, the consistent findings of altered SCFA levels in various retinal diseases underscore their potential importance in disease pathogenesis. These findings pave the way for future research exploring the therapeutic potential of modulating SCFA levels, either through direct supplementation or indirectly by targeting the gut microbiota, to influence the course of retinal diseases.

### 3.3. Effects of SCFA Supplementation on Retinal Health

Several studies in this review investigated the effects of short-chain fatty acid (SCFA) supplementation, particularly sodium butyrate, on various aspects of retinal health using animal models. Huang et al. (2023) [36] examined the impact of oral sodium butyrate administration in a mouse model of type 1 diabetes (T1D) induced by Streptozotocin. These STZ-induced mice mimic DR’s thinning and inflammation, which butyrate counters, much like laser–CNV models replicate AMD’s neovascular damage. The study found that butyrate supplementation significantly ameliorated retinal thinning, specifically in the inner and middle retinal layers, and inhibited microglial activation, a key component of retinal inflammation. Furthermore, butyrate treatment improved retinal function, as evidenced by improved electroretinography (ERG) parameters, including reduced implicit times of a- and b-waves and increased amplitudes of a-waves, b-waves, and oscillatory potentials (OPs). These findings suggest that butyrate may protect against diabetes-induced retinal structural and functional deficits. In addition to this, in the study performed by Shen et al. (2022) [34], it was found that both alpha-linolenic acid (ALA) and linoleic acid (LA) treatments were able to prevent the retinal thinning that was observed in the diabetic group, especially in the ganglion cell layer (GCL). A significant reduction was observed in the number of cells in the GCL, inner nuclear layer (INL) and outer nuclear layer (ONL) in the diabetic group, as well as an increase in this number in the ALA and LA groups, especially in the GCL, which returned to near that of the control. Here, ALA and LA spurred SCFA production through gut microbiota modulation, not direct retinal action, aligning with our gut–retina focus.

Rowan et al. (2017) [29] demonstrated that a low-glycemic diet in 60 mice naturally elevated butyrate and acetate levels, curbing photoreceptor damage in an AMD-like model—mirroring the structural benefits Huang et al. (2023) [36] saw with butyrate in diabetic mice. Unlike direct supplementation, this dietary approach leans on microbiota shifts, akin to Zhang et al.’s (2023) [35] metformin-driven SCFA boost, suggesting diverse routes to the same retinal rescue. These findings collectively underscore SCFAs’ protective potential, whether delivered straight or coaxed from the gut.

Xiao et al. (2020) [30] investigated the effects of sodium butyrate on choroidal neovascularization (CNV), a hallmark of neovascular age-related macular degeneration (nAMD), using both in vitro and in vivo models. In cultured human umbilical vein endothelial cells (HUVECs), butyrate inhibited cell proliferation and tube formation in a dose-dependent manner. In a laser-induced CNV mouse model, intravitreal injection of sodium butyrate significantly reduced CNV lesion size. These anti-angiogenic effects were associated with the upregulation of thioredoxin-interacting protein (TXNIP) and downregulation of vascular endothelial growth factor receptor 2 (VEGFR2), suggesting that butyrate may inhibit CNV by modulating the TXNIP/VEGFR2 pathway. This was further supported by Dos Reis et al. (2022) [33], who developed sodium butyrate-loaded nanoparticles coated with chitosan (NaBu-loaded nanoparticles/CS) and demonstrated their antiangiogenic activity in a chick chorioallantoic membrane (CAM) assay. While intravitreal injection of free sodium butyrate caused retinal damage in rats, the NaBu-loaded nanoparticles/CS were well-tolerated and showed no signs of retinal toxicity in both in vitro and in vivo models, highlighting the importance of a controlled delivery system. This damage stemmed from free butyrate’s direct injection, unlike the nanoparticle delivery that safely curbed angiogenesis. Shifting to gut-driven effects, Zhang et al. (2023) [35] showed that oral metformin administration can have beneficial effects on the retina by reducing the CNV lesion size in a laser-induced CNV mouse model. This effect was associated with changes in the gut microbiota, specifically an increase in the abundance of *Akkermansia* and *Bifidobacterium* genera, as well as an increase in the levels of fecal butyrate and propionate.

In addition to the direct effects on the retina, some studies also found that sodium butyrate supplementation was associated with systemic improvements. Huang et al. (2023) [36] reported that butyrate treatment in diabetic mice reduced blood glucose levels, food intake, and water consumption, although it did not significantly affect body weight. Moreover, butyrate supplementation enhanced the expression of tight junction proteins (ZO-1 and Occludin) in the small intestine, suggesting an improvement in intestinal barrier function. The potential systemic effects of butyrate highlight the interconnectedness of the gut–retina axis and suggest that interventions targeting the gut microbiota may have broader implications for overall health in the context of retinal diseases. Table 2 summarizes the results of SCFA supplementation in experimental studies.

### 3.4. Mechanisms of the Gut–Retina Axis: Evidence from the Reviewed Studies

SCFA receptors, particularly GPR41, GPR43, and GPR109A, are known to mediate many of the physiological effects of SCFAs. Zhang et al. (2023) [35] provide indirect evidence for the involvement of these receptors by demonstrating that the protective effects of metformin against CNV were microbiome-dependent. Metformin can increase the abundance of SCFA-producing bacteria, such as *Akkermansia* and *Bifidobacterium*, potentially leading to increased SCFA production. This, in turn, could activate SCFA receptors in the retina, although this was not directly measured. In addition, Chen et al. (2021) [31] showed that SCFAs mitigate LPS-induced uveitis, likely via systemic immune modulation, though direct retinal penetration remains unconfirmed. Similarly, the review by Zhang et al. (2023) [35] highlights the importance of these receptors in mediating the effects of SCFAs in various tissues, including the gut.

Sodium butyrate is a well-established inhibitor of histone deacetylases (HDACs). By blocking HDAC activity, sodium butyrate promotes histone acetylation, which relaxes chromatin structure and enhances the expression of genes tied to anti-inflammatory and neuroprotective pathways [40]. In the retina, this mechanism could dampen inflammatory responses and bolster neuronal survival, as seen in studies where butyrate curbs microglial activation and supports retinal function in diabetic models. This epigenetic modulation underscores why butyrate supplementation shows promise beyond simple metabolic effects, offering a molecular bridge between gut microbial activity and retinal homeostasis. Dos Reis et al. (2022) [33] demonstrated the antiangiogenic effects of butyrate-loaded nanoparticles, suggesting that HDAC inhibition might play a role in this context. Huang et al. (2023) [36] observed an upregulation of tight junction proteins in the small intestine following butyrate treatment, an effect that has been linked to HDAC inhibition in other studies.

Several studies highlight the importance of the gut microbiota in mediating the effects of SCFAs and other interventions. Zhang et al. [35] showed that metformin’s effects were abolished in germ-free mice, and FMT from metformin-treated mice replicated the protective effects. Huang et al. [36] demonstrated that butyrate supplementation altered gut microbiota composition and increased systemic SCFA levels, while also improving intestinal barrier function. These findings suggest that the gut microbiota plays a crucial role in the gut–retina axis, potentially by modulating SCFA production, influencing systemic inflammation, and altering the levels of other metabolites, such as bile acids. The studies by Vergroesen et al. [38], Qin et al. [39], Baldi et al. [37], Shen et al. [34] and Chen et al. [32] further support this notion by demonstrating altered gut microbiota composition in patients with glaucoma, nAMD, and DR, respectively.

There is a direct link between SCFAs and the modulation of inflammatory processes in the retina. Chen et al. [31] showed that SCFAs, including butyrate, inhibited the production of pro-inflammatory cytokines (IL-6, TNF-alpha) and chemokines (CXCL1, CXCL12) by LPS-stimulated retinal astrocytes in vitro. This suggests a direct anti-inflammatory effect of SCFAs on retinal cells. Huang et al. [36] observed a reduction in microglial activation, a marker of retinal inflammation, in diabetic mice treated with butyrate. Moreover, Shen et al. [34] observed that treatment with both ALA and LA was associated with a decrease in the expression of pro-inflammatory cytokines. Collectively, these findings strongly suggest that the beneficial effects of SCFAs on retinal health are, at least in part, mediated by their ability to suppress retinal inflammation. SCFAs might also bolster retinal health as mitochondrial energy substrates, a role hinted at by butyrate’s metabolic support in high-energy retinal cells.

## 4. Discussion

This systematic review aimed to synthesize the current evidence on the relationship between short-chain fatty acids (SCFAs), the gut microbiota, and retinal diseases, with a particular focus on the gut–retina axis. We sought to answer the following question: what is the role of SCFAs, as modulators of the gut microbiota, in the pathogenesis and potential treatment of retinal diseases?

### 4.1. The Impact of Gut Microbiota on SCFA Production and Ocular Health

A consistent pattern observed across our included studies was the alteration of gut microbiota composition in conditions like diabetic retinopathy (DR), neovascular age-related macular degeneration (nAMD), and glaucoma. These alterations were often characterized by a decreased abundance of beneficial SCFA-producing bacteria. For instance, glaucoma patients exhibited a lower abundance of butyrate-producing taxa such as *Butyrivibrio* and *Clostridium* (Vergroesen et al. [38]), while DR patients showed a reduction in *Butyricicoccus*, in the *Ruminococcus torques* group (Qin et al. [39]). Similarly, nAMD patients demonstrated decreased levels of genera such as *Bacteroides*, *Faecalibacterium*, and *Lachnospira* (Baldi et al. [37]). These findings align with the broader literature on gut dysbiosis in various diseases, as reviewed by Serban et al. (2023) [41]. They emphasize the potential importance of butyrate-producing bacteria in maintaining retinal health, consistent with the established role of butyrate in promoting intestinal barrier integrity and exerting anti-inflammatory effects (Zhang et al. [35]). The observed increase in potentially pro-inflammatory bacteria, such as *Peptococcus* in DR (Qin et al. [39]) and *Escherichia-Shigella* in nAMD (Baldi et al. [37]), further supports the link between gut dysbiosis and retinal inflammation. The observation by Zhao et al. (2022) [42] that *Bifidobacterium* can promote retinal ganglion cell survival and modulate retinal glial cell responses after optic nerve injury underscores the potential of specific bacterial strains to exert neuroprotective effects. Rowan et al. (2017) [29] further illustrate this dynamic, showing that a low-glycemic diet in mice ramps up *Bacteroides* and *Prevotella*, boosting butyrate and acetate to shield against AMD-like retinal damage—echoing Zhao et al.’s (2022) [42] findings on *Bifidobacterium*’s neuroprotective role. This dietary nudge aligns with Baldi et al.’s (2024) [37] micronutrient-driven SCFA shifts, suggesting that microbial composition is not just a bystander but a key player in ocular outcomes.

Further supporting the gut–retina axis, Labetoulle et al. (2024) [43] describe how gut dysbiosis, characterized by reduced *Faecalibacterium prausnitzii* and increased *Escherichia coli*, may contribute to ocular surface disorders by disrupting intestinal barrier integrity and promoting systemic inflammation via translocation of bacterial products, such as lipopolysaccharides. This concept is further reinforced by Xue et al. (2021) [44], who emphasize the role of the gut microbiota in modulating the host’s immune response, particularly the balance between T-helper cell subsets (Th1, Th2, Th17) and regulatory T cells (Tregs). They propose that a decrease in SCFA-producing bacteria can impair Treg cell differentiation and function, potentially promoting a Th17-driven inflammatory response that could contribute to ocular diseases like autoimmune uveitis, age-related macular degeneration, and glaucoma. Adding to this body of evidence, Liu et al. (2024) [45] demonstrated that antibiotic-induced gut dysbiosis and germ-free conditions disrupted circadian gene expression, barrier integrity, nerve density, and immune cell activity in the mouse cornea. Notably, they showed that SCFA supplementation significantly restored corneal integrity in mice with antibiotic-induced gut dysbiosis, highlighting the crucial role of the gut microbiota and its metabolites in maintaining corneal homeostasis. Moreover, they found that SCFA receptors are expressed in various cell types in the cornea, further supporting the idea that SCFAs have a direct effect on the ocular surface. Thus, the dysbiotic changes observed in our included studies, along with the findings of Labetoulle et al. (2024) [43], Xue et al. (2021) [44], and Liu et al. (2024) [45], suggest a complex interplay between gut microbiota composition, SCFA production, immune modulation, and ocular health.

### 4.2. SCFA Levels in Retinal Diseases

Concordant with the dysbiotic changes in gut microbiota, our review identified alterations in SCFA levels in individuals with retinal diseases. Notably, several studies reported decreased levels of key SCFAs, including butyrate, propionate, and acetate, in the plasma or feces of patients with DR or nAMD [34,36,37,39]. These findings are consistent with the decreased abundance of SCFA-producing bacteria observed in these conditions. The study by Chen et al. [32], however, found increased levels of SCFAs in both the fecal and blood samples of patients with glaucoma, highlighting potential disease-specific variations in SCFA metabolism. Zhang et al. [35] provide a comprehensive overview of SCFA production, absorption, and metabolism, highlighting their diverse roles in host health. The observed reductions in SCFA levels in DR and nAMD may have significant implications for retinal health, given the known anti-inflammatory, neuroprotective, and barrier-enhancing effects of SCFAs, particularly butyrate. For instance, SCFAs have been shown to inhibit HDAC activity, which plays a crucial role in retinal cell differentiation and gene expression (Ferreira et al., 2016 [46]). Moreover, SCFAs can modulate immune responses by interacting with specific GPCRs, such as GPR41, GPR43, and GPR109A. These receptors are expressed in various tissues, including the intestine and immune cells, and their activation by SCFAs can influence inflammatory pathways relevant to retinal diseases. Wang et al. (2017) [47] identified a potential role for histone H1 modification and autophagy in diabetic retinopathy, suggesting that epigenetic mechanisms may be involved in the retinal response to metabolic disturbances, which can be modulated by gut microbiota. This aligns with the broader understanding of SCFAs’ ability to act as HDAC inhibitors, potentially influencing gene expression and cellular processes in the retina, as suggested by our analysis. The protective role of SCFAs in ocular inflammation is further supported by Wu et al. (2024) [48], who showed that SCFAs, specifically butyrate, inhibit corneal inflammatory responses to TLR ligands in mice via the G-protein coupled receptor 43 (GPR43). Scuderi et al. (2022) [49] further highlight the crucial role of SCFAs in modulating host immune responses and regulating gene expression via mechanisms such as histone deacetylase inhibition and G-protein-coupled receptor activation. Specifically, Scuderi et al. emphasize the capacity of SCFAs to influence diverse immune cells and signaling pathways through interactions with receptors like PXR, VDR, LXRs, FXR, and TGR5, offering a mechanistic framework that likely extends to the gut–retina axis and the pathogenesis of ocular diseases, as supported by our study.

### 4.3. SCFA Supplementation as a Therapeutic Target

The studies included in our review provide compelling evidence for the therapeutic potential of targeting the gut–retina axis, particularly through SCFA supplementation. Oral administration of sodium butyrate was shown to ameliorate retinal thinning, inhibit microglial activation, and improve ERG parameters in diabetic mice (Huang et al. [36]). These findings are supported by studies demonstrating the anti-angiogenic effects of butyrate in CNV models (Xiao et al. [30]) and the efficacy of butyrate-loaded nanoparticles in reducing neovascularization (Dos Reis et al. [33]). The beneficial effects of butyrate may be mediated, in part, through its ability to upregulate neurotrophic factors like GDNF and BDNF (Wu et al., 2008) [50], which are crucial for neuronal survival and function. In line with this, Shen et al. [34] showed that supplementation with LA and ALA was able to increase the levels of SCFAs, also ameliorating retinal damage in diabetic mice. Furthermore, the study by Zhang et al. [35] demonstrated that oral metformin, a widely used antidiabetic drug, inhibited CNV in mice by modulating the gut microbiome, including an increase in SCFA-producing *Akkermansia* and *Bifidobacterium*. The protective effects of metformin were abolished in germ-free mice, highlighting the critical role of the gut microbiota in mediating its effects on the retina. These findings align with the broader literature on the therapeutic potential of SCFAs, reviewed by Serban et al. (2023) [41], and suggest that interventions aimed at restoring a healthy gut microbiota and optimizing SCFA production, such as dietary modifications or targeted supplementation, may hold promise for the prevention and treatment of retinal diseases. In addition, the study conducted by Baldi et al. [37] found that micronutrient supplementation was associated with an improvement in visual acuity, although there were no changes in the gut microbiome composition. Lastly, while focused on metabolic disorders, the study by Gao et al. (2019) [51] demonstrated that butyrate supplementation can improve glucose intolerance, modulate gut dysbiosis, and influence lipid metabolism pathways in high-fat-diet-fed mice. These findings are relevant to our review as they highlight the systemic benefits of butyrate, which may also extend to the improvement of the retinal microenvironment. Rowan et al. (2017) [29] add a twist, showing that a low-glycemic diet in mice naturally lifts butyrate and acetate, cutting photoreceptor loss in an AMD model—much like Huang et al.’s (2023) [36] butyrate-driven retinal rescue in diabetic mice. This dietary path complements Zhang et al.’s (2023) [35] metformin boost to SCFA-producers like *Akkermansia*, hinting that whether through supplements, drugs, or diet, SCFAs pack a therapeutic punch for the retina.

### 4.4. Limitataions of Included Studies

The studies included in this review provide valuable insights into the relationship between SCFAs, the gut microbiota, and retinal diseases, but it is important to acknowledge their limitations. Several studies employed animal models, such as laser-induced CNV in mice and STZ-induced diabetic mice, to investigate the effects of SCFA supplementation or modulation of the gut microbiome. While these models offer controlled experimental conditions, their translatability to human retinal diseases may be limited. For instance, the laser-induced CNV model primarily recapitulates the neovascular aspects of AMD, and the STZ-induced diabetes model, while mimicking some features of diabetic retinopathy, does not fully capture the complex and multifactorial nature of human diabetes and its complications. Studies relying on dietary or drug interventions [34,35] struggle to isolate SCFAs as the sole drivers of retinal outcomes, a caveat direct butyrate trials sidestep. Some studies, like Dos Reis et al.’s [33] with only 18 rats, relied on smaller sample sizes that may limit statistical power, yet this is offset by larger cohorts in our review, such as Rowan et al.’s [29] with 60 mice, Qin et al.’s [39] with 161 humans, and Vergroesen et al.’s [38] with 1472 humans. Incorporating these heftier samples strengthens the generalizability of our findings, though the variability in scale underscores the need for further large-scale validation. Furthermore, inherent differences in gut microbiota composition between rodents and humans may influence the observed effects, warranting careful interpretation when extrapolating findings to humans.

In addition to the limitations inherent in animal models, the human studies included in this review were primarily observational and cross-sectional in design, which precludes the establishment of causal relationships between gut dysbiosis, SCFA alterations, and retinal disease development. Furthermore, relatively small sample sizes in some studies, including both human and animal studies, may reduce the statistical power and generalizability of the findings. Moreover, while efforts were made to control for potential confounding factors such as age, sex, and BMI in some studies, the influence of unmeasured variables, including detailed dietary habits and lifestyle factors, cannot be entirely ruled out.

Future research employing longitudinal designs, larger sample sizes, and comprehensive assessments of potential confounders, coupled with interventional studies, will be crucial to validate these findings and elucidate the causal relationships between the gut microbiota, SCFAs, and retinal diseases.

### 4.5. Limitataions of Our Study

This systematic review has several limitations that should be considered when interpreting the findings. First of all, we did not perform a formal risk of bias assessment using standardized tools like SYRCLE (for animal studies) and RoB 2 (for human studies). This decision was made due to the heterogeneity of study designs and the exploratory nature of this review. However, this may affect the ability to weigh the strength of evidence.

Secondly, the heterogeneity of our included studies limits the generalizability of our findings. The studies in this review varied considerably in terms of study design (animal models, human observational studies, clinical trial), species (mice, rats, humans), interventions (SCFA supplementation, dietary interventions, metformin), outcome measures, and methodological approaches. The majority of the studies investigating the effects of SCFA supplementation were conducted in animal models. Although these studies provide valuable information, the findings may not be directly translatable to humans due to differences in physiology, gut microbiota composition, and disease pathogenesis. Lastly, most of the included studies, both animal and human, had small sample sizes. This might further limit the statistical power to detect significant effects and could affect the generalizability of our conclusions.

## 5. Conclusions

This systematic review provides compelling evidence for a significant association between the gut microbiota, short-chain fatty acids (SCFAs), and the pathogenesis of retinal diseases. Our analysis reveals that individuals with conditions such as diabetic retinopathy, age-related macular degeneration, and glaucoma often exhibit gut dysbiosis, characterized by a reduced abundance of beneficial SCFA-producing bacteria and alterations in SCFA profiles. Experimental studies further demonstrate that interventions aimed at modulating the gut microbiota, including the administration of SCFAs like butyrate, can ameliorate retinal pathology and improve retinal function in animal models. These beneficial effects are likely mediated through a combination of anti-inflammatory, anti-angiogenic, and neuroprotective mechanisms, involving pathways such as HDAC inhibition and specific GPCR activation. Taken together, these findings underscore the potential of the gut–retina axis as a novel therapeutic target, suggesting that strategies to restore gut microbial balance and enhance SCFA production may offer new avenues for preventing and treating retinal diseases. 

## Figures and Tables

**Table 2:** SCFA supplementation in experimental studies. LA = linoleic acid; ALA = alpha-linolenic acid; NaBu = sodium butyrate; ERG = electroretinography; CNV = choroidal neovascularization.

Study	SCFA(s) Investigated	Model (Species)	Route of Administration	Dosage/Duration	Key Retinal Findings	Other Findings
Xiao et al., 2020 [30]	Sodium butyrate	Laser-induced CNV (mice), HUVECs (in vitro)	Intravitreal injection, in vitro	In vivo: Not specified in abstract; In vitro: various	Reduced CNV lesion size, inhibited HUVEC proliferation and tube formation, upregulated TXNIP, downregulated VEGFR2	
Chen et al., 2021 [31]	SCFAs (acetate, propionate, butyrate)	LPS-induced uveitis (mice)	Intraperitoneal injection	In vivo: 500 mg/kg, in vitro: 1, 5, 10 mM	Reduced production of IL-6, TNF-alfa, CXCL1, and CXCL12 by LPS-stimulated RACs in vitro; reduced severity of uveitis in vivo	Enhanced antigen presenting ability of RACs in vitro; reduced immune cell migration in vitro
Dos Reis et al., 2022 [33]	Sodium butyrate	Wistar rats, ARPE-19 cells, CAM	Intravitreal injection, in vitro	34.4 µg/mL (nanoparticles)	Nanoparticles: no retinal toxicity, antiangiogenic in CAM assay; Free NaBu: retinal damage	Nanoparticles: controlled release of NaBu for 35 days
Shen et al., 2022 [34]	LA and ALA	STZ-induced T1DM (rats)	Intraperitoneal injection	100 μg/day every other day for 3 weeks	Prevented retinal thinning, reduced cell number reduction	Improved lipid profiles; modulated gut microbiota; reduced inflammation
Zhang et al., 2023 [35]	Oral metformin (indirectly increased butyrate and propionate)	Laser-induced CNV (mice)	Oral gavage	300 mg/kg	Reduced CNV lesion size, decreased Iba1+ macrophages/microglia around the lesion.	Altered gut microbiome composition; increased fecal SCFAs; modulated RPE/choroid gene expression
Huang et al., 2023 [36]	Sodium butyrate	STZ-induced T1DM (mice)	Oral gavage	500 mg/kg daily for 12 weeks	Ameliorated retinal thinning (inner/middle layers), inhibited microglial activation, improved ERG parameters	Reduced blood glucose, food, and water consumption; enhanced tight junction protein expression in the small intestine

## Data Availability

The original contributions presented in the study are included in the article. Further inquiries can be directed to the corresponding author.

## Abbreviations

The following abbreviations are used in this manuscript:

SCFA	Short-chain fatty acid
AMD	Age-related macular degeneration
DR	Diabetic retinopathy
GPCRs	G-protein coupled receptors
HDAC	Histone Deacetylase Inhibitors
CNV	Choroidal neovascularization
HUVEC	Humans umbilical vein endothelial cells
ALA	Alpha-linolenic acid
LA	Linoleic acid
GCL	Ganglion cell layer
ERG	Electroretinography
NaBu	Sodium butyrate
CAM	Chorioallantoic membrane
